# Effects of *Talinum triangulare* leaf flavonoid extract on streptozotocin‐induced hyperglycemia and associated complications in rats

**DOI:** 10.1002/fsn3.765

**Published:** 2018-10-11

**Authors:** Olarewaju M. Oluba, Feyikemi D. Adebiyi, Ajibola A. Dada, Adeyinka A. Ajayi, Kayode E. Adebisi, Sunday J. Josiah, Adewale A. Odutuga

**Affiliations:** ^1^ Food Safety and Toxicology Research Unit Environment and Technology Research Cluster Department of Biological Sciences College of Science and Engineering Landmark University Omu Aran Kwara State Nigeria; ^2^ Department of Chemical Sciences Joseph Ayo Babalola University Ikeji Arakeji Osun State Nigeria; ^3^ Biochemistry Unit Department of Bioscience and Biotechnology College of Pure and Applied Sciences Kwara State University Ilorin Kwara State Nigeria; ^4^ Department of Biochemistry College of Basic Medical Sciences Igbinedion University Okada Edo State Nigeria

**Keywords:** diabetic dyslipidemia, flavonoid extract, HMG‐CoA reductase, hyperglycemia, *Talinum triangulare*

## Abstract

*Talinum triangulare* leaf flavonoid extract (TTFE) was evaluated for its effects on streptozotocin‐hyperglycemia and associated complications especially as it relates to dyslipidemia, lipid peroxidation, and renal dysfunction in rats. Two normoglycemic rat groups designated: control (administered distilled water) and control + TTFE (administered 10 mg/kg b.w. TTFE) and two streptozotocin‐induced (STZ) diabetic rat groups designated: STZ‐control (administered distilled water) and STZ + TTFE (administered 10 mg/kg TTFE). The treatment was given orally once daily for 21 consecutive days. Body weight and insulin concentration showed significant improvement while blood glucose, uric acid, creatinine, and total bilirubin concentrations were significantly reduced in diabetic rats administered TTFE compared to diabetic untreated rats. Furthermore, triglycerides, total cholesterol, LDL‐cholesterol, and malondialdehyde concentrations were significantly lowered in diabetic rats administered TTFE compared with diabetic untreated rats. Key enzymes involved in carbohydrate breakdown and cholesterol synthesis, α‐amylase and 3‐hydroxy‐3‐methylglutaryl‐CoA (HMG‐CoA) reductase, respectively, were significantly inhibited in TTFE‐treated diabetic rats compared to diabetic control. Results presented in this study suggest that administration of TTFE for 21 days normalized STZ‐induced hyperglycemia and its associated dyslipidemia by a mechanism involving inhibition of α‐amylase and HMG‐CoA reductase activities, respectively, in rats.

## 1 INTRODUCTION

Experimental and epidemiological studies have most often shown a close association between blood glucose concentration and cardiovascular disease (Coutinho, Gerstein, Wang, & Yusuf, 1999). About 40%–80% of diabetic subjects have been reported to present with an increased risk of cardiovascular disease (Cheung & Li, 2012). Similar to that, individuals presenting with cardiovascular‐related disorders also demonstrate some levels of impairment in glucose tolerance (Tominaga et al., 1999). Therefore, efforts aimed at controlling hyperglycemia must also include treatment of associated hyperlipidemia in order to reduce cardiovascular events in diabetes (Goldberg et al., 2005).

Insulin‐regulated stimulation of the sympathetic nervous system is most often accompanied by an enhanced glucose delivery and utilization in peripheral tissues (Goodpaster & Wolf, 2004). In addition, hepatic glucose mobilization and the release of free fatty acids in adipose tissue as well as the synthesis of muscle protein are all coordinately regulated by insulin (Jung, Lee, Park, Kang, & Choi, 2006). However, varying multifactorial associations are involved in the development of insulin resistance, ranging from genetics, environmental to obesity (Karpe, Dickmann, & Frayn, 2011).

Reports from literature have shown that some pharmacological agents used in the treatment of cardiovascular‐related disorders such as statins have demonstrated promising potential in reducing vascular event outcome in patients with diabetes and hypertension (Deedwania, 2011). Therefore, there has been a growing interest in the search for plant‐derived hypoglycemic agents. Flavonoids are a diverse group of naturally occurring polyphenolic compounds in plants which are major components of human and animal diets (Eleazu, Obianuju, Eleazu, & Kalu, 2017). Plant bioflavonoids have been demonstrated to possess varying health‐promoting effects including anticancer, antioxidant, antimicrobial, antihypertensive, antiulcer, antipyretic, and antidiabetic properties (Auger et al., 2005; Mojzisova, Petrasova, & Koprovicova, 1999; Oboh et al., 2012). Plant bioflavonoid has been demonstrated to be effective as antihyperglycemic and antihyperlipidemic agents in animal models of diabetes (Achi, Ohaeri, Ijeh, & Eleazu, 2017). For example, quercetin and fisetin which belong to the flavonoid family have been demonstrated to lower blood glucose level through their inhibitory activity on mammalian alpha‐amylase (Etxeberria, de la Garza, Campión, Martinez, & Milagro, 2012).

There are about 500 species of the genus Talinum worldwide used as an aphrodisiac (Adithya, Sasikumar, Krishnakumar, Lakshmi, & Christabel, 2012). Most often, the leaf is made into powder and mixed with boiled milk in the traditional treatment of diabetes (Thalapaneni, Chidambaram, Ellappan, Sabapathi, & Mandal, 2008; Thalapaneni, Sabapathi, Ansari, & Mandal, 2011). The ethanolic extract of *Talinum triangulare* leaves has been reported to inhibit the production of free radical that often potentiate membrane lipid peroxidation (Adefegha & Oboh, 2011; Liao, Chai, Wang, Chen, & Tsai, 2015). Similar to that, the methanolic extract of *Talinum portulacifolium* leaves was also reported to inhibit intestinal alpha‐glucosidase activity in rats (Thalapaneni et al., 2008) while leave extracts of *T. portulacifolium* obtained with hexane and water were reported to show antihyperglycemic and antioxidant effects (Babu et al., 2009). However, literature on the potential health benefits of the leaf flavonoid extract of *T. triangulare* is scarce. This present study was designed to evaluate the biochemical actions of *T. triangulare* leaf flavonoid extract on diabetic hyperglycemia and its associated complications in streptozotocin‐induced diabetic rats.

## 2 MATERIALS AND METHODS

### 2.1 Chemical and reagents

(+)‐Catechin and Folin–Ciocalteu reagent were purchased from Sigma Chem. Co. (USA), AB‐8 adsorption resin (0.3–1.25 mm, Nankai University Chemical Plant, Tianjin, China), Streptozotocin (Sigma, USA), commercial reagent kits for determination of alanine aminotransferase (ALT), aspartate aminotransferase (AST), triglycerides (TG), total protein (BCA protein assay kit), superoxide dismutase (SOD), and glutathione peroxidase (GPx) were products of Randox Laboratories Ltd (Crumlin, County Antrim, UK). All other chemicals were of analytical grade.

### 2.2 Plants material

Fresh aerial leaves of *T. triangulare* were obtained from the Teaching and Research Farm of the Department of Crop/Soil Science, Joseph Ayo Babalola University, Ikeji Arakeji, Nigeria, and were botanically identified by Mr Kehinde Oyebanji, a taxonomist in the Department of Crop Science, Joseph Ayo Babalola University, Ikeji Arakeji, Osun State, Nigeria.

### 2.3 Extract preparation

Fresh aerial leaves of *T. triangulare* were air‐dried at 27°C for 7 days and milled into powder with a mechanical grinder. The powdered material (200 g) was extracted by maceration with 400 ml of distilled water at room temperature. After filtration and evaporation of the solvent under reduced pressure, the resulting residue was re‐extracted thrice with distilled water. The filtrate was pooled together, filtered with Whatman number 1 filter paper. The filtrate was then concentrated at 50°C using a Speed Vac (Model 7811001; Labconco, USA) and stored until use.

### 2.4 Phytochemical screening

The extract was subjected to phytochemical analysis according to the methods described by Brain and Turner (1975).

### 2.5 Quantitative estimation of flavonoids

Total flavonoid content was determined following the procedure described by Kosalec, Bakmaz, Pepeljnjak, and Vladimir‐Knezevic (2004) using catechin as a standard.

#### 2.5.1 Microwave‐assisted extraction of *Talinum triangulare* flavonoids

Extraction of the flavonoid content of *T. triangulare* extract was achieved following the procedure previously described by Ghharekhani, Rafiee, Ghorbani, and Jafari (2009).

#### 2.5.2 Purification of *Talinum triangulare* flavonoid extract

The extracted flavonoid purified in a 400 × 2.5 (cm i.d.) column packed activated AB‐8 resin. The extract was poured into the column and allowed to absorb into the column for 10 min. Bound carbohydrates were removed by washing the column thoroughly with distilled water and then eluted with 65% ethanol to remove the flavonoids. The resulting flavonoid‐rich eluent was concentrated using a rotary evaporator at 4°C before being stored at −4°C for further analysis.

### 2.6 Animals treatment

Forty (40) female adult Swiss albino rats weighing (200.2–210.15 g) were purchased from University of Ibadan, Ibadan, Nigeria. They were kept in filter top cages in an environmentally controlled room (26.0 ± 2.6°C, 50%–60% relative humidity with a 12‐hr day and night cycle). They were fed commercially rat pellet and tap water ad libitum throughout the duration of the experiment and were treated according to the international guidelines for the care and use of laboratory animals (ILAR, 1985). The animals were kept for 2 weeks before commencement of the experiment to acclimatize.

### 2.7 Induction of diabetes in rats

The animals were administered 50 mg/kg bw freshly prepared STZ in 0.1 M citrate buffer (pH 4.5) intraperitoneally. Forty‐eight hours following STZ administration, tail blood sample was obtained for glucose estimation. Animals with post‐STZ glucose concentration >250 mg/dl were considered diabetic and used for further study.

### 2.8 Animal groupings and treatments

A total of 40 rats (comprising twenty diabetic and twenty nondiabetic) divided into four groups (*n* = 10) on the basis of their weight and designated:

*Control*: Consisted of normal rats administered 2 ml distilled water
*Control* + *TTFE*: Consisted of nondiabetic rats administered 10 mg/kg b.w. *T. triangulare* leaf flavonoid extract
*STZ‐control*: Consisted of STZ‐diabetic rats administered 2 ml of distilled water
*STZ* + *TTFE*: Consisted of STZ‐diabetic rats administered 10 mg/kg body weight *T. triangulare* leaf flavonoid extract.

Treatment was administered by oral gavage once daily for 21 consecutive days. Daily food and water intakes were recorded for each group while body weight gain was recorded weekly. After 21‐day treatment, the animals were fasted overnight and their fasting blood glucose concentration estimated from their tail blood samples before been euthanized and then sacrificed by cervical dislocation. Upon sacrifice, 2 ml blood sample from each animal was collected into a plain sample bottle, liver and lung were quickly excised, blotted on tissue paper and kept in phosphate buffer saline at −4°C for further analysis.

### 2.9 Oral glucose tolerance test

Oral glucose tolerance test (OGTT) was carried out after the 21‐day treatment period following the procedures described by Chaturvedi, George, Milinganyo, and Tripathi (2004). In brief, rats in both control and control + TTFE groups were fasted overnight following which blood was obtained from the tail vein of each rat at 0, 30, 60, 90, and 120 min, respectively, after oral administration of 3 g/kg glucose for blood glucose determination.

### 2.10 Sample preparation

Blood samples collected in plain tubes were left standing for 3 hr to clot and then centrifuged at 3000 x *g* for 10 min at 4°C. Serum from the centrifuged blood sample was collected by suction using pasture pipette into sterile plain sample bottles and stored at −4°C for further analysis. The liver homogenate was prepared by weighing 2 g of cleaned tissue and homogenized using 0.1 M phosphate buffer after which it was centrifuged at 5,000 rpm (4°C) for 10 min to obtain the supernatant which was subsequently used as tissue homogenate.

### 2.11 Biochemical analysis

Glucose concentration was determined using the glucose oxidase method of Trinder (1969). Serum insulin was estimated by a double‐antibody radioimmunoassay, using a Synergy HTX Multi‐Mode Microplate Reader (Biotech Instruments Inc, USA). Serum uric acid concentration was estimated following the modified Beale colorimetric method as described by Buchanan, Isdale, and Rose (1965). Urea and creatinine were determined according to the methods of Richterich and Kuffer (1973) and Blass, Thiebert, and Lam (1974), respectively. Bilirubin estimation was by Michaelsson, Nosslin, and Sjölin (1965) method. Malondialdehyde (MDA), as an index of lipid peroxidation, was estimated by the method of Buege and Aust (1978) while superoxide dismutase (SOD) and glutathione peroxidase (GPx) activities were determined using Randox commercial kits following the manufacturer's instructions. Triglyceride concentration was determined following the protocol described by Carr, Andressen, and Rudel (1993). Total cholesterol concentration was estimated according to the method of Allain, Chan, Poon, Richard, and Fu (1974), and HDL‐cholesterol according to Warmick, Benderson, and Albers (1982). LDL‐cholesterol concentration was estimated using the method of Princen, van Poppel, Vogelezang, Buytenhek, and Kok (1992).

### 2.12 Estimation of alpha‐amylase and 3‐hydroxy‐3‐methylglutaryl‐CoA reductase activities

Alpha‐amylase activity was determined using the microplate‐based starch‐iodide method previously detailed by Xiao, Ni, Kai, and Chen (2013). The ratio of the concentration of 3‐hydroxy‐3‐methylglutaryl‐CoA to mevalonate in the liver was used as a measure of the activity of HMG‐CoA reductase (Rao & Ramakrishnan, 1975).

### 2.13 Statistical analysis

The data are presented as means ± *SEM* of ten determinations. The mean values of control and test groups were compared using one‐way analysis of variance (ANOVA) and Duncan Multiple Range Test (DMRT) performed using GraphPad prism software (version 6.05). *p* < 0.05 was considered to be significant.

## 3 RESULTS

### 3.1 Phytochemical screening and flavonoid content

Phytochemical screening of the aqueous extract of *T. triangulare* leaf showed that tannins, phenol, flavonoids, alkaloids, and anthraquinone were present. The total flavonoid yield was 161.2 μg/mg.

### 3.2 Food intake, water intake, and body weight

After the 21 days treatment period, the quantity of food consumed and water intake in diabetic rat groups (STZ‐control and STZ + TTFE) was significantly (*p* < 0.05) higher compared to nondiabetic rat groups (control and TTFE + control). However, food and water intakes were significantly (*p* < 0.05) reduced in diabetic animals administered *T. triangulare* flavonoid extract (STZ + TTFE) compared with diabetic rats administered distilled water (STZ‐control) (Table 1). Body weight gain significantly (*p* < 0.05) lower in STZ‐induced diabetic rat groups (STZ‐control and STZ + TTFE) compared to nondiabetic control groups (control and control + TTFE). However, there was a significant increase in body weight (34.1 ± 3.2) in diabetic rats administered *T. triangulare* flavonoid extract (STZ + TTFE) compared with diabetic untreated rats (12.9 ± 2.1) (Table 1).

**Table 1:** Food intake, water intake, and body weight gain of streptozotocin‐induced diabetic rats administered *Talinum triangulare* leaf flavonoid extract over a period of 21 days

Parameters	Treatment groups			
	Control	Control + TTFE	STZ‐control	STZ + TTFE
Food intake (g rat^−1^ day^−1^)	29.6 ± 2.9^a^	25.3 ± 7.7^a^	48.9 ± 7.7^c^	32.5 ± 5.5^b^
Water intake (ml rat^−1^ day^−1^)	42.5 ± 3.8^a^	46.1 ± 5.3^a^	46.1 ± 8.3^c^	78.1 ± 5.5^b^
Body weight gain (g)	40.2 ± 2.5^b^	40.9 ± 5.1^b^	12.9 ± 2.1^a^	34.1 ± 3.2^b^

Note

Values are expressed as means ± *SEM* of five determinations. Values in the same rows carrying different superscript are statistically significant (*p* < 0.05).

### 3.3 Effects on blood glucose and serum insulin

**Figure 1:**
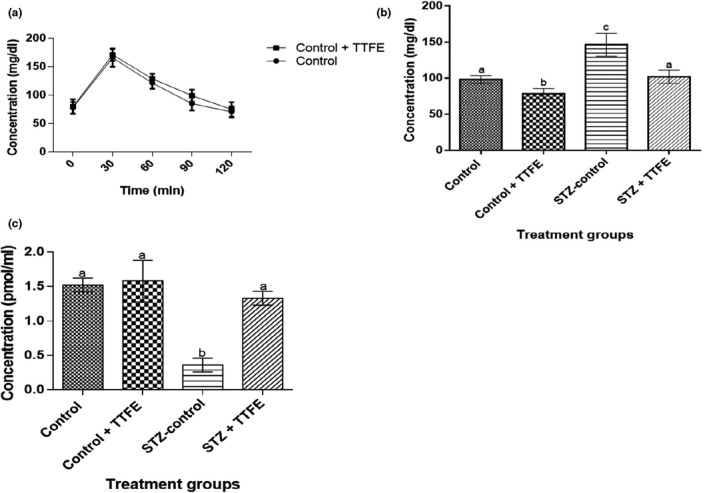
(a) Oral blood glucose concentration following a 21‐day administration of flavonoid extract of *Talinum triangulare*, (b) and (c) effects of flavonoids extract of *T. triangulare* on blood glucose concentration and insulin level, respectively, in streptozotocin‐diabetic rats over a period of 21 days. Results are represented as means ± *SEM* of five determinations. Bars with different alphabet are statistically significant (*p* < 0.05)

Response to oral glucose challenge was similar in rats administered 10 mg/kg TTFE for 21 days and normal rats (Figure 1a). Blood glucose concentration was significantly (*p* < 0.05) higher in STZ‐diabetic rats (STZ‐control and STZ + TTFE) compared to the nondiabetic rat groups (control and TTFE + control) (Figure 1b). However, following treatment with TTFE, blood glucose concentration was significantly (*p* < 0.05) reduced in STZ‐diabetic rats compared to diabetic untreated rats (administered distilled water). A 20% and 30% reduction in blood glucose concentration was observed in nondiabetic and diabetic rats, respectively, administered TTFE. Serum insulin concentration was significantly lower (*p* < 0.05) in the diabetic rat groups (STZ‐control and STZ + TTFE) compared to nondiabetic rat groups (control and control + TTFE) (Figure 1c). However, administration of TTFE resulted in significant (*p* < 0.05) improvement in serum insulin levels in STZ‐diabetic rats compared to diabetic untreated rats.

### 3.4 Effects on kidney function parameters

**Figure 2:**
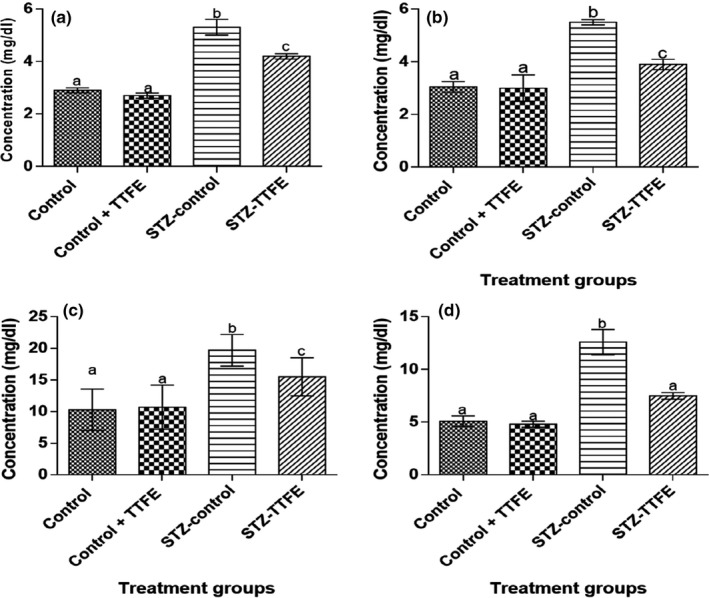
Serum uric acid (a), urea (b), creatinine (c), and total bilirubin (d) concentrations in streptozotocin‐diabetic rats treated with *Talinum triangulare* leaf flavonoid extract for 21 days. Results are represented as means ± *SEM* of 10 determinations. Bars with different alphabets are statistically significant (*p* < 0.05)

Serum uric acid, urea, creatinine, and bilirubin concentrations were significantly (*p* < 0.05) lower in diabetic rats administered TTFE (STZ + TTFE) compared to diabetic untreated rats (STZ‐control) (Figure 2). The observed uric acid, urea, creatinine, and bilirubin levels between the nondiabetic rat groups (control and control + TTFE) were not significantly (*p* > 0.05) different.

### 3.5 Effects on lipid peroxidation and antioxidant enzymes

**Figure 3:**
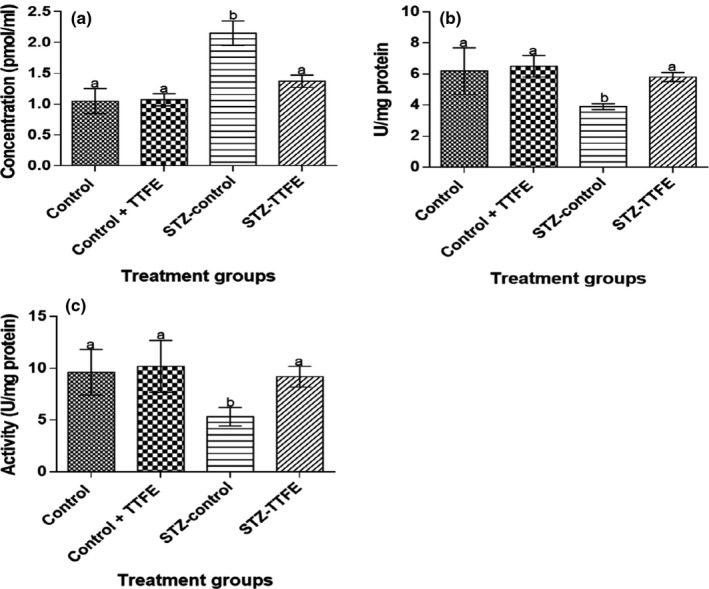
Malondialdehyde concentration (a), glutathione peroxidase (b), and superoxide dismutase (c) in streptozotocin‐diabetic rats treated with *Talinum triangulare* leaf flavonoid extract for 21 days. Results are represented as means ± *SEM* of 10 determinations. Bars with different alphabets are statistically significant (*p* < 0.05)

Serum MDA concentration was significantly (*p* < 0.05) higher the STZ‐diabetic rat groups (STZ‐control and STZ + TTFE) compared to the nondiabetic rat groups (control and control + TTFE). Serum superoxide dismutase (SOD) and glutathione peroxidase (GPx) activities were significantly (*p* < 0.05) higher in nondiabetic rats administered TTFE (control + TTFE) compared to nondiabetic rats administered distilled water (control). Similar to that, diabetic rats administered TTFE (STZ + TTFE) had significantly higher (*p* < 0.05) SOD and GPx activities compared to diabetic rats administered distilled water (STZ‐control) (Figure 3).

### 3.6 Effects on lipid profile and hepatic HMG‐CoA reductase activity

**Figure 4:**
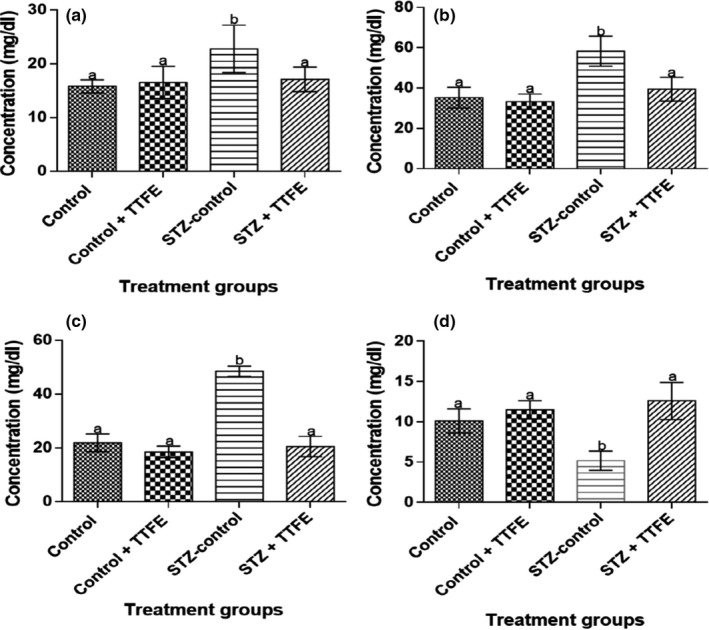
Serum triglyceride (a), total cholesterol (b), low‐density lipoprotein cholesterol (c), and high‐density lipoprotein cholesterol (d) in streptozotocin‐diabetic rats treated with *Talinum triangulare* leaf flavonoid extract for 21 days. Results are represented as means ± *SEM* of 10 determinations. Bars with different alphabets are statistically significant (*p* < 0.05)

**Figure 5:**
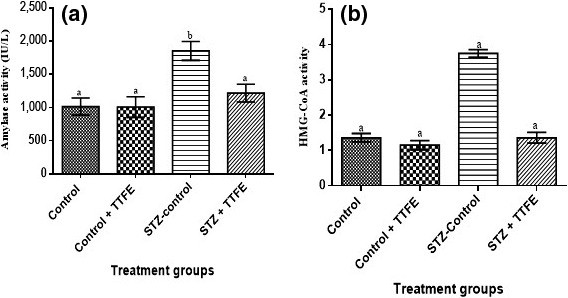
Serum alpha‐amylase (a) and 3‐hydroxyl methylglutaryl‐CoA reductase (b) activities in streptozotocin‐diabetic rats treated with *Talinum triangulare* leaf flavonoid extract. Values are expressed as means ± *SEM* of 10 determinations. Bars with different alphabet are statistically significant (*p* < 0.05)

Serum triglycerides, total cholesterol, and LDL‐cholesterol concentrations were significantly lower (*p* < 0.05) in diabetic rats administered TTFE compared to diabetic untreated rats (STZ‐control) (Figure 4). Alpha‐amylase and HMG‐CoA activities were significantly lower in rat groups administered TTFE (control + TTFE and STZ + TTFE) compared to nondiabetic and diabetic rats administered distilled water (control and STZ‐control) (Figure 5).

## 4 DISCUSSION

The present study demonstrated that administration of *T. triangulare* leaf flavonoid extract (10 mg/kg) to STZ‐diabetic rats for 21 days led to a significant improvement in body weight, blood glucose, and serum insulin concentrations. Serum uric acid, urea, creatinine, and total bilirubin concentrations were also significantly reduced giving an indication of improved kidney function. Significant reduction in serum MDA with a corresponding increase in SOD and GPx activities was also observed in diabetic rats administered 10 mg/kg *T. triangulare* leaf flavonoid extract. Similar to that, serum triglyceride, total cholesterol, and low‐density lipoprotein cholesterol concentrations were also significantly reduced. In addition, serum alpha‐amylase (a key enzyme regulating the release of glucose from ingested carbohydrates into the blood streams) and HMG‐CoA reductase (the rate‐limiting enzyme in the synthesis of cholesterol) activities were significantly inhibited in STZ‐diabetic rats administered treated with 10 mg/kg *T. triangulare* leaf flavonoid extract for 21 days.

The oral glucose tolerance test gives a good index of insulin release, as well as its sensitivity to blood glucose level (Rhee et al., 2010). Therefore, it is a reliable marker for evaluating an individual's capacity to utilize ingested glucose over a given period of time (Eleazu et al., 2017). Under normal condition blood glucose concentration at 2 hr following oral glucose loading is about 110 mg/dl. However, the level rises above 110 mg/dl in the condition of impaired glucose tolerance due to insulin insufficiency or insensitivity, culminating in impaired uptake and utilization of glucose by the muscle and adipose tissues. Thus, medicinal plants exhibiting antidiabetic activity by mechanisms involving improvement in glucose tolerance (Achi et al., 2017). Therefore, the findings from this study which indicated that rats administered 10 mg/kg *T. triangulare* leaf flavonoid extract for 21 days were well able to tolerate oral glucose challenge in a manner similar to normal rats is of significance.

Induction of diabetes in rodents by streptozotocin is due to its cytotoxic action on β‐cells of the pancreas (Szkudelski, 2001; Wohaieb & Godin, 1987). In accordance with previous report (Al‐Awwadi et al., 2004), the classical clinical manifestations of diabetes type I including weight loss, increased food and water intakes and reduced insulin concentrations in rats administered STZ were also observed in the present study. However, the observed decrease in body weight due to STZ‐induced diabetes in this study could be an indication of muscle wasting as a consequence of the loss or degradation of structural proteins. Muscle wasting usually results from the gluconeogenic synthesis of glucose from lipid and proteinaceous materials as a compensatory strategy for the nonavailability of glucose (in the diabetic state) for utilization as an energy source (Sathish Sekar, Sivagnanam, & Subramanian, 2005). In addition, the present study demonstrated that oral administration of 10 mg/kg *T. triangulare* leaf flavonoid extract for 21 days significantly prevented the hyperglycemic action of STZ in rats.

Furthermore, data presented in this study showed that administration of *T. triangulare* leaf flavonoid extract at 10 mg/kg prevented the attendant diabetic complications in STZ‐diabetic rats. Plasma insulin concentration showed significant improvement in STZ‐rats administered *T. triangulare* leaf flavonoid extract. Thus, providing further justification for the antihyperglycemic action of this extract in STZ‐diabetic rats. Several plants bioflavonoids have been demonstrated to exert multiple actions on the synthesis and release of insulin from β‐cells. Jung et al. (2006) showed that citrus bioflavonoid supplementation in mice led to a significant increase in plasma insulin secretion. Similar to that, reports by Prince and Kamalakkannan (2006) demonstrated a significant increase in plasma insulin concentration in diabetic rats following prolonged rutin administration. Thus, the ability of the flavonoid extract of *T. triangulare* leaf to increase plasma insulin concentration as demonstrated in this study could provide a basis for its observed hypoglycemic action in diabetic rats. Insulin plays a major biochemical role in stimulating the uptake of glucose by different cells of the body for the production of energy (Cummings et al., 2004).

Data presented showed increased serum uric acid concentration in diabetic rats. This may be attributable in part to derangement in some metabolic processes in diabetic state prominent among which is increased xanthine oxidase activity, lipid peroxidation, hypertriglyceridemia as well as high cholesterol concentration (Anwar & Meki, 2003). Moreover, the breakdown of muscle protein as a compensatory strategy for glucose synthesis through gluconeogenesis culminating in muscle wasting and increased release of purine, which is the main source of uric acid are a possible explanation to the high uric acid levels in diabetes (Anwar & Meki, 2003). Plasma uric acid, urea, and creatinine levels were substantially reduced in STZ‐rats following 21‐day administration of 10 mg/kg *T. triangulare* leaf flavonoid extract. Polyphenolic flavonoids in cherries were reported to have a unique reputation as antigout and anti‐inflammatory agents (Jacob et al., 2003). Elevated serum urea and creatinine concentrations are established markers of renal dysfunction in diabetic hyperglycemia (Almadal & Vilstrup, 1988). Moreso, previous studies have indicated a causal relationship between blood glucose concentration and end‐stage renal damage (de Zeeuw et al., 2004).

Diabetes is most often characterized by derangement in lipid metabolism. Uncontrolled type 1 diabetes mellitus is associated with increased serum total cholesterol as well as LDL‐cholesterol concentrations with decrease HDL‐cholesterol concentration which contribute to the coronary artery disease (Goodpaster & Wolf, 2004; Maron et al., 2003). The action of streptozotocin on pancreatic beta cells is thought to be partly mediated through the production of free radicals such as H_2_O_2_, O_2_, and HO^.^ (Szkudelski, 2001; Wohaieb & Godin, 1987). The observed increase in plasma MDA level in STZ‐diabetic rats in this study presumably could predispose the tissue to lipid peroxidation unless adequate amounts of antioxidants are present. In addition, increased lipid peroxide level in diabetes may be attributed to the increased glycation of protein which might themselves act as a source of free radicals in diabetes mellitus (Marzouk & Karoui, 2013).

Data from this study indicated a positive association between lipid peroxide (MDA) and glucose concentration. Thus, the observed decreases in plasma MDA concentrations vis‐a‐vis increases in endogenous activities of GPx and SOD following the administration of *T*. *triangulare* leaf flavonoid extract in STZ‐diabetic rats in this study give credence to the antioxidative potential of the extract, especially in diabetic state. The observed reduction in serum triglycerides and cholesterol levels in this study is in agreement with the reports from previous studies (Auger et al., 2005; Goodpaster & Wolf, 2004; Maron et al., 2003). These authors postulated that the hypotriglyceridemic action plant bioflavonoids could have been mediated through inhibition of fatty acid synthesis (Auger et al., 2005; Goodpaster & Wolf, 2004). The increase in triglyceride concentration in STZ‐diabetic rats in this study could be attributed to decrease in insulin secretion culminating in reduced tissue glucose utilization and the consequent mobilization of fatty acids from adipose tissue as a compensatory measure. Adipose tissue free fatty acids are mobilized for energy production and the resulting excess fatty acid are accumulated in the liver, and converted to triglyceride (Karpe et al., 2011). In addition, insulin concentration has been reported to be responsible for controlling the number of LDL receptors (Duvillard et al., 2003), hence, insulin deficiency as observed in the diabetic untreated rats in this study may have led to a reduction in the number of LDL receptors. With respect to the cholesterol‐lowering property of polyphenolic compounds, it has been suggested that some constituents of *T. triangulare* flavonoid extract may act as inhibitors for some enzymes such as hydroxymethyl glutaryl CoA reductase, which participates in cholesterol synthesis (Theriault et al., 2000). Consistent with this idea, data from this study showed that *T. triangulare* leaf flavonoid extract inhibited hepatic HMG‐CoA reductase activity in STZ‐diabetic rats. An increase in this ratio indicates inhibition of cholesterogenesis while a decrease suggests enhanced cholesterogenesis. Thus, the observed antilipidemic action of the flavonoid extract of *T. triangulare* demonstrated in this study could have involved its ability to inhibit hepatic HMG‐CoA reductase, the rate limiting enzyme in cholesterol synthesis.

Data generated from this study demonstrated the induction of α‐amylase activity in STZ‐diabetic rats thus contributing to an overall 15.8% increase in glycemia in STZ‐diabetic rats. However, an over 50% inhibition was observed in diabetic rats administered *T. triangulare* flavonoids extract. The consequent down‐regulation of α‐amylase activity in concert with increased insulin secretion could have contributed to the near 40% reduction in blood glucose concentration in the diabetic rats observed in this study. The inhibitory action of *T. triangulare* flavonoids on the α‐amylase activity could have contributed to the reduction in carbohydrate hydrolysis and absorption in the intestine thus leading to a decrease in serum glucose levels (Oboh et al., 2012).

## 5 CONCLUSION

Results presented in this study suggest that administration of TTFE for 21 days normalized STZ‐induced hyperglycemia and its associated dyslipidemia by mechanisms involving enhanced plasma insulin secretion which could possibly stimulate cellular glucose uptake; inhibition of α‐amylase activity thus regulating the release of glucose into the blood and regulation of hepatic lipid synthesis via inhibition of HMG‐CoA reductase activity in rats.

## CONFLICT OF INTEREST

Authors declare that there exists no conflict of interest.

## ETHICAL APPROVAL

All applicable international, national, and/or institutional guidelines for the care and use of animals were followed.
